# 

**Published:** 1876-12

**Authors:** 


### *BOOK NOTICES.*

Books make the best holiday presents. The most useful book a family can possess—the Bible excepted—is an encyclopedia. It contains articles on almost every subject of interest to men. "Zell's Encyclopedia and Universal Dictionary," in two quarto volumes, contains more articles than other encyclopedias of ten to sixteen volumes. While equal to, and in many respects superior, to other works, its price is only $32. Published by Baker, Davis & Co., Philadelphia.

"Living too Fast." By "Oliver Optic." Published by Lee & Shepard, Boston.

The story records the experience of a young bank officer, who had the weakness to commit a crime in order to obtain the means to gratify his extravagant desires and those of his young wife, whom he adored.

The story is well written and exceedingly interesting, but we fear the author has impaired the force of the "moral" in his desire to please the reader with a favorable ending. The train of circumstances that shielded Glasswood from the penalty of his crime are, at least, improbable. The course of "fast livers" and the results of "fast living" are truthfully portrayed in the career of this young man.

"Vine and Olive" is the fifth volume of the second series of "Young America Abroad." In this volume are related the travels and adventures in Spain and Portugal of the students belonging to the *Academy Squadron* The plot is well laid, and the lectures by the professors are useful as well as entertaining. Its author—"Oliver Optic"—is well known to juvenile readers, and he is a general favorite. Published by Lee & Shepard, Boston, Mass.

**A.** PAGE.

A Word for Ourselves....................483
Airing Chambers........................ 14
A Good Digestion....................... 47
A Man.................................. 62
Air and Exercise....................... 71
Averting Disease....................... 80
Abuse of Purgative Medicines...........129
A Wife Worth Having....................174
An Easy Death..........................214
An Explanation.........................250
Announcement...........................250
A Good Fire Insurance Company..........253
Antidote for Poisons...................256
A Chat About Health....................277
A New Duty.............................280
A Simple Remedy........................290
A Co-operative Store in New York.......296
An Article by Dr. Hall.................299
A Model Schoolroom.....................318
A Remedy for Every Ill..................334
A Question of Food................343, 394
A Gossip with Women....................347
A Rank Offence.........................379
An Erring Witness......................439
A Bit of Humor.........................440
A New Enterprise.......................441

**B.**

Bodily Carriage........................ 31
Bread Poison........................... 90
Bushels of Pills....................... 93
Balance of Population.................. 94
Body and Mind..........................125
Burying Alive..........................143
Buckwheat Cakes........................515
Bad Cooking............................247
Bowel Complaints of Children......380, 454
Book Reviews...........................444
Brandy and Throat Disease..............484

**C.**

Common Sense........................... 42
Cancer and Constipation................ 76
Changing Flannel....................... 78
Corns Cured............................ 85
Cancer................................. 99
Civilization and Longevity.............514
Curative Power of Hot Springs..........120
Chronic Nasal Catarrh..................122
Counterfeits...........................124
Cold Philosophy........................134
Coffee Drinking........................141
Cause of Consumption...................159
Clerical Marriages.....................162
Cold Water Bathing.....................186
Curious Table of Figures...............219
Communication..........................252
Cholera Infantum.......................281
Cold Baths.............................289
Contracting Diseases...................305
Constipation...........................309
Crinoline Dangers......................311
Consumption Localities.................316
Cooked Meats...........................393
Causes of Fever and Ague...............419

**D.**

Death in Doors......................... 13
Disease and Crime...................... 28
Dangers of Oratory..................... 61
Do You Mean What You Say...............100
Diarrhœa...............................161
Donation Parties..................182, 274
Dyspepsia..............................505
Dentistry..............................228
Dying Nations..........................234
Death's Summer Campaign................245
Destructive Agencies...................279
Depopulation...........................307
Deaths in New York.....................315
Drunken Doctors........................338
Dr. Dewey's Enterprise.................442

**E.**

Early Breakfast........................ 56
Eyesight...............................167
Eating and Exercise....................273
Excelsior of Agriculture...............508

**F.**

Failing Eyesight....................... 58
Family Order...........................193
Fly Exterminator.......................212
Food...................................282
Fat Folks..............................294
Fruit Seasons..........................314
From Laurie Todd's Note Book...........315
Furnaces...............................422
"Fire on the Hearth"...................442

**G.**

Genuine Enterprise..................... 7
Getting Married........................ 18
Gluttony........................... 28, 89
Growing Old Happily.................... 57
Groundless Fears.......................104
Gymnasiums.............................177
Good Night (Poetry)....................260
Gloved to Death........................281
Grand Sequences........................358
Going to the South.....................478

**H.**

Hall's Journal for 1776................ 1
Heat and Cold.......................... 8
How Much to Sleep...................... 72
Honesty and Push....................... 98
How it is Done.........................126
Households Contrasted..................132
Hearty Suppers.........................157
Hydrophobia............................168
Human Growth...........................196
How to Get Sick........................210
How a Doctor Looks.....................213
How to Prevent Colds...................218
Health and Capital.....................248
Homes for Invalids.....................249
Hydropathy.............................283
Health of Cities.......................485
Hammer On..............................463

**I.**

Interesting to All..................... 10
Instinct of Appetite................... 44
In the Twilight (Poetry)...............128
Insanity...............................215
In a Hurry.............................221
Inverted Toe Nails.....................224
Infants and Air........................234
In Poor Health.........................246
In the Long Run........................283
Intussusception........................317
Inconsiderations.......................486

**K.**

Keeping the Teeth Clean................482

**L.** PAGE.

Lifting Machine........................ [illegible]

Life on Railroads........................ 37

Liquor License........................ 88

Leguminous Food........................ 97

Leaning Providence........................235

Lost (Poetry)........................303

Late Suppers........................375

Living on Excilement........................473

**M.**

Material for Men........................165
Medicines........................101
Medical Principles........................221
Miasm and Malaria........................227
Make Home Happy........................310
Malaria........................399
Monomaniacs........................425
My Home in New England........................487
Making a Fire........................506

**N.**

Nothing to Do........................ 17
New York Husbands........................ 53
Nutrition and Stimulation........................ 91
Neglecting Colds........................180
New Potato........................196
Nelaton's Suppository........................223
New Publications........................255
Notice to Dyspeptics........................444

**O.**

Over Eating........................ 80
Our Country........................226
Obituary........................243
Out of Evil Good........................265
Our Defences........................319
Oldboy Brothers' Nearest Relatives......487

**P.**

Popular Prejudices........................ 3
Poisonous Milk........................197
Portrait (Dr. W. W. Hall)........................241
Paper for Water Closets........................302
Poisonous Rooms........................308
Physical Culture........................312
Position in Reading........................316
Profanity........................337
Periodical Literature........................455
Poisonous Soap........................421
Pure Water........................423
Pertaining to Insurance..........443, 397, 341
Public Schools........................461
Physical Training........................465
Pork as Food........................516

**Q.**

Question of Morals........................ 92

**R.**

Reading Aloud........................ 83
Rattlesnakes and their Bites........................348
Ragusa........................354
Roserl........................401, 445

**S.**

Sleeping in Church........................ 11
Suicide........................ 36
Sleep, Delicious........................ 46
Sea Voyages........................ 48
Sleeplessness........................ 49
Spring Diseases........................ 58
Sources of Trouble and Joy........................ 62
Successful........................ 63
Sabbath Hygiene........................ 65
Scarlet Fever........................105
Surgical Instruments........................143
Sedentary Habits........................144

PAGE.

Surgical Diseases........................151

School Dangers........................166

Some Health Principles........................169

Sleep of Children........................171

Sympathy for the Erring........................230

Stomach and Liver........................232

Sociability........................277

School Outrages........................279

Save the Babies........................292

See, Saw, Marjorie Daw........................297

Spirometer........................301

Saratoga Springs........................390

Similarity of Individuals and Social Organisms........................400

Sir Astley Cooper's Bath........................480

Self-Medication........................526

**T.**

Tight Shoes........................ 26
True Hospitality........................ 77
Teeth of Children........................ 28
The Best Tooth Wash........................ 85
The Service of Chemistry to Pharmacy...108
The Health Food Company......434, 109, 386
The Physician........................145
The Aim of Life........................154
The Modern Man........................156
The Three Glories........................157
Trouble Kills........................172
The Marriage Relation........................175
Throat and Lungs........................200
Toe Nails, Inverted........................202
The Panacea........................224
The Last Wish........................229
The Air We Breathe........................229
True Temperance........................231
Time Table........................236
The Atheist (Poetry)........................251
Treatment of Cancer........................254
The Poetry of Poverty........................257
Themes for the Thoughtful........................270
Taking Medicine........................278
The Grahamite Theory........................291
The Mystery at Silver Cliff............321, 363
The Poor Babies........................331
The Hours Most Fatal to Life........................474
The Battle and the Burthens of Life......475
The Hardest Mode to Die........................477
The Lungs........................483
The Human Hair........................519
Taking Cold........................512

**U.**

Unearthly Localities........................335

**V.**

Variety of Diet........................338, 382, 426

**W.**

Wholesome Food........................ 9
Winter Diseases........................ 11
Water Cure........................ 79
Winter Railroading........................176
Waste and Want........................202
Wearing Flannel........................217
War........................260
Whiskey Doctors........................274
Ways to Drunkenness........................319
Weak Lungs........................333
Wakefulness........................373
Wearing Glasses........................377
Wholesome Flour........................429
Wheat and Flour........................433
Warming Houses........................527
Winter Rules........................511

